# Modulation of HIV-1-host interaction: role of the Vpu accessory protein

**DOI:** 10.1186/1742-4690-7-114

**Published:** 2010-12-22

**Authors:** Mathieu Dubé, Mariana G Bego, Catherine Paquay, Éric A Cohen

**Affiliations:** 1 Laboratory of Human Retrovirology, Institut de Recherches Cliniques de Montréal (IRCM), 110, Avenue des Pins Ouest, Montreal, Quebec, Canada H2W 1R7; 2 Department of Microbiology and Immunology, Université de Montréal, Montreal, Quebec, Canada

## Abstract

Viral protein U (Vpu) is a type 1 membrane-associated accessory protein that is unique to human immunodeficiency virus type 1 (HIV-1) and a subset of related simian immunodeficiency virus (SIV). The Vpu protein encoded by HIV-1 is associated with two primary functions during the viral life cycle. First, it contributes to HIV-1-induced CD4 receptor downregulation by mediating the proteasomal degradation of newly synthesized CD4 molecules in the endoplasmic reticulum (ER). Second, it enhances the release of progeny virions from infected cells by antagonizing Tetherin, an interferon (IFN)-regulated host restriction factor that directly cross-links virions on host cell-surface. This review will mostly focus on recent advances on the role of Vpu in CD4 downregulation and Tetherin antagonism and will discuss how these two functions may have impacted primate immunodeficiency virus cross-species transmission and the emergence of pandemic strain of HIV-1.

## Introduction

HIV-1 interaction with host target cells is complex with nearly every step of the virus infection cycle relying on the recruitment of cellular proteins and basic machineries by viral proteins [1]. For instance, the Tat regulatory protein recruits the pTEFb complex during viral transcription to enhance host RNA polymerase II processivity and promote efficient elongation of viral transcripts (reviewed in [2]). Similarly, the p6 domain of the Gag structural protein interacts with the ESCRT complex during viral assembly to direct the budding of progeny virions (reviewed in [3]). Recent discoveries have shed light on an additional level of complexity involving host proteins that provide considerable resistance to infection by HIV-1 and other viruses via cell-autonomous mechanisms that are likely part of the antiviral innate immune response. As a virus which induces a persistent infection, HIV-1 has evolved countermeasures to overcome the antiviral activity of these host factors, also called restriction factors, mainly through the activities of a set of viral accessory proteins that include the Vif, Vpr, Vpu and Nef proteins. These accessory proteins, which have been recently the subject of intense research and progress, represent one of the defining features of primate immunodeficiency viruses. They are not commonly found in other retroviruses and as such are likely to play a key role in HIV-1 pathogenesis. Overall, it is becoming increasingly clear that the function of these non-enzymatic viral proteins is to modulate the cellular environment within infected cells to promote efficient viral replication, transmission and evasion from innate and acquired immunity (for recent reviews [4,5]). In this review, we will focus on the recent progress in our understanding of the functions and mode of action of the HIV-1 Vpu accessory protein and relate these to the pathogenesis of the virus as well as the emergence of pandemic HIV-1 strains. Furthermore, we will highlight some important questions for the future.

## The *vpu *gene product

Vpu was initially identified as the product of an open reading frame (ORF), referred as the U ORF (initially all HIV-1 ORFs were designated by alphabetical letters) located between the first exon of the *tat *and *env *genes of HIV-1 [6,7]. The *vpu *gene is present in the genome of HIV-1 but is absent from HIV-2 and other related SIVs, such as SIV from sooty mangabey (SIVsmm) and SIV from rhesus macaques (SIVmac) [6,7]. Structural homologues have been detected in SIV from chimpanzee (SIVcpz), the precursor of HIV-1, and in SIVs from the mona monkey (*Cervicopithecus mona*; SIVmon), the greater spot-nosed monkey (Cercopithecus nictitans; SIVgsn), the mustached monkey (*Cercopithecus cephus*; SIVmus) and more recently in Dent's mona monkey (*Cercopitheus mona denti*; SIVden) and gorilla (*Gorilla gorilla*; SIVgor) [8-13].

**Figure 1:**
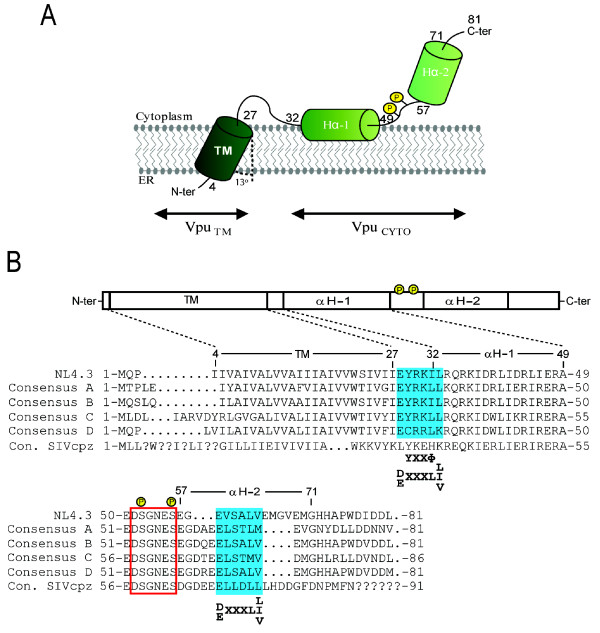
**Schematic representations of Vpu**. (A) Predicted secondary and tertiary structure of Vpu showing the N-terminal transmembrane domain (TM) and the two α-helices of the cytoplasmic (CYTO) domain. The numbers indicate amino acid positions of the NL4.3 prototypical Vpu allele. In both panels, yellow circles represent phosphorylated serine residues (S52 and S56) sites. The 13° tilt angle of the TM domain is indicated. (B) Vpu topology with the corresponding HIV-1/SIVcpz *Ptt *Vpu consensus sequences (HIV sequence database, http://www.hiv.lanl.gov). Question marks indicate residues with no consensus available. The red box indicates the conserved sequences recognized by β-TrCP. The blue boxes highlight areas containing putative trafficking signals shown below. X and Φ correspond to variable and hydrophobic amino-acid residues, respectively. αH: α-helix.

The Vpu protein encoded by HIV-1 is a 77-86 amino-acids membrane-associated protein capable of homo-oligomerization [14]. The protein is translated from a Rev-dependent bicistronic mRNA, which also encodes the viral envelope glycoprotein (Env), suggesting that expression of Vpu and Env are coordinated during HIV-1 infection [15]. The protein is predicted to have a short luminal N-terminal domain (3-12 amino acids), a single transmembrane (TM) spanning domain that also serves as an uncleaved signal peptide (23 amino acids) and a charged C-terminal hydrophilic domain of 47-59 residues that extends into the cytoplasm [14,16] (Figure 1A). While the crystal structure of the entire Vpu protein has yet to be solved, the molecular structure of the N-terminal domain (residues 2-30) has been determined by nuclear magnetic resonance (NMR) and found to contain a TM α-helix spanning residues 8 to 25 with an average tilt angle of 13 degrees [17,18]. Interestingly, modeling as well as biochemical and genetic evidence have suggested that the TM domain is critical for Vpu oligomerization and that a pentameric structure for the TM domain would be optimal for the formation of an ion channel [19,20]. In that regard, several studies have suggested that Vpu, like the M2 protein of influenza, may have an ion channel activity (for a recent review [21]). However, whether the ion channel activity of Vpu is required for Vpu function is still controversial. NMR analysis of the cytosolic domain, on the other hand, revealed that this part of the protein comprises two α-helical regions interconnected by a flexible loop containing a highly conserved sequence (DSGNES) [16,22,23], which includes a pair of serine residues (S52 and S56) that are phosphorylated by casein kinase II [24-27] (Figure 1).

HIV-1 Vpu proteins encoded by subtype B strains appear to be largely expressed on intracellular membranes, which correspond to the ER, the *trans *Golgi network (TGN) as well as endosomal compartments, but no accumulation of the protein is readily detected at the cell surface [28-30]. Unlike the better-studied HIV-1 subtype B Vpu proteins, subtype C and SIVcpz Vpu alleles fused to EGFP were reported to be transported to the plasma membrane [31-33]. Interestingly, as depicted in Figure 1B, amino acid sequence analysis of the cytosolic domain of HIV-1 Vpu reveals the presence of putative trafficking signals that harbor a degree of amino acid variation among Vpu alleles from different subtypes [34]. These trafficking signals include: 1) an overlapping tyrosine (YXXΦ where Φ designs a hydrophobic residue) and an acidic/dileucine based ([D/E]XXXL[L/I/V]) sorting motifs in the hinge region between the TM anchor and the cytosolic domain, normally implicated in endocytosis as well as in the targeting of TM proteins to lysosomes and lysosome-related organelles [35]; 2) another acidic/dileucine sorting signal, [D/E]XXXL[L/I/V], in the second α-helix of the Vpu cytoplasmic tail [32] (Figure 1B). The fact that several laboratory-adapted strains and primary isolates of HIV-1 harbor *vpu *genes with polymorphism at the level of these putative trafficking signals [29,34] raises the possibility that regulation of Vpu subcellular localization and perhaps biological activities may indeed confer the virus a selective advantage in some physiological conditions.

Studies mostly performed with Vpu originating from subgroup B laboratory-adapted strains (NL4-3, BH10) have established two main functions during infection of HIV-1 target cells in tissue culture systems. First, Vpu induces a rapid degradation of newly synthesized CD4 receptor molecules in the ER via the ubiquitin-proteasome system [36,37]. In addition to its effect on CD4 catabolism, Vpu promotes the release of progeny virions from HIV-1-infected human cells [7,26,28,38-40] by counteracting Tetherin (also designated BST2, CD317 and HM1.24), a host restriction factor that strongly inhibits the release of virions from the host cell surface [41,42].

## Role of Vpu in HIV-1-induced CD4 receptor downregulation

### Expression of CD4 molecules at the surface of HIV-1 infected cells is detrimental to efficient viral replication and spread

The process of HIV-1 entry into target cells begins with the binding of the viral envelope glycoprotein gp120 to both the CD4 receptor and one of the chemokine co-receptors, CXCR4 or CCR5 [43]. Despite the critical role played by the CD4 receptor during viral entry, it is well established that an early and lasting effect of infection is the downregulation of the CD4 receptor from the host cell surface. In fact, it appears that once viral entry has occurred, continuous expression of the CD4 receptor may be detrimental to efficient viral replication and spread. Early work on this issue has shown that newly synthesized CD4 molecules are capable of retaining the Env precursor proteins in the ER through their high Env binding affinity, therefore preventing transport and processing of mature Env products, gp120 and gp41, to the site of virus assembly [44-47]. Additionally, expression of CD4 at the cell surface promotes superinfection of cells by cell-free and cell-associated viruses [48] and can interfere with the efficient release of infectious progeny virions from the cell surface [49-54]. While the disadvantageous effect of CD4 on the release and infectivity of cell-free virus is well established, it is unclear whether the expression of CD4 at the cell surface of infected cells also impedes cell-to-cell viral transmission through virological synapses, a mode of propagation that is believed to promote efficient viral dissemination [55,56]. Despite its compact genome, HIV-1 devotes two accessory proteins, Nef and Vpu, to the task of suppressing expression of its primary receptor. Early in infection, Nef removes mature CD4 molecules that are already present at the cell surface by enhancing their endocytosis by a pathway involving clathrin and AP2 [57-59] followed by delivery of internalized CD4 to the multivesicular body pathway for eventual degradation in the lysosomes [60,61]. In contrast, Vpu, which is expressed late during the virus life cycle, acts on newly synthesized CD4 molecules in the ER and as such counteracts their effects in the early biosynthetic pathway [62]. This functional convergence, involving two viral proteins acting on CD4 molecules located in different cellular compartments and operating by distinct mechanisms, implies that cell surface CD4 downregulation must play an important role for HIV-1 replication and propagation.

### Vpu hijacks the host ubiquitin machinery to target CD4 for proteasomal degradation

**Figure 2:**
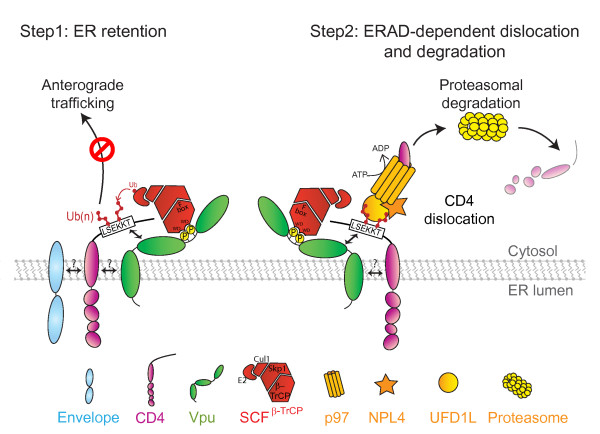
**Model of Vpu-mediated CD4 degradation**. First, Vpu retains CD4 in the ER through TM domains interactions; formation of Env/CD4 complexes could contribute to this retention. In addition, CD4 and Vpu also interact through their cytosolic domains. The minimal region of the CD4 cytoplasmic tail conferring Vpu sensitivity was mapped to the region 414-LSEKKT-419. Recruitment of the SCF^β-TrCP ^E3 ubiquitin ligase complex by Vpu is mediated by interactions of phosphoserines in Vpu and the WD boxes of β-TrCP. Interactions between Vpu and CD4 result in the *trans*-ubiquination of the cytosolic tail of CD4 on lysine, serine and threonine residues. These ubiquitination events might further contribute to CD4 retention in the ER but, importantly, target CD4 for degradation by the cytosolic proteasome. This targeting involves a dislocation step mediated by the p97-UFD1L-NPL4 complex, a critical component of ERAD. This complex recognizes K48-linked polyubiquitinated chains on the cytosolic tail of CD4 through the UFD1L co-factor. The p97 protein via its ATPase activity subsequently directs the dislocation of CD4 across the ER membrane where the receptor becomes readily accessible for proteasomal degradation.

The degradation of CD4 mediated by Vpu involves multiple steps that are thought to be initiated by the physical binding of Vpu to the cytoplasmic tail of CD4 in the ER. Mutational and deletion analyses of CD4 have delineated a domain of the molecule, encompassing residues 414 and 419 (LSEKKT) as well as an α-helix located in the membrane proximal region of the viral receptor cytosolic region, that are required for Vpu binding and CD4 degradation [63-67] (Figure 2). The domain of Vpu that is interacting with the cytosolic region of CD4 is still not precisely defined but previous studies have shown that these binding determinants are likely to be present in the cytoplasmic region of the protein [68]. In support of this finding, a mutant of Vpu that harbored a TM domain with a randomized primary sequence was still able to bind CD4 and mediate its degradation as well as its wild-type (WT) counterpart [69]. Furthermore, mutational analysis of the Vpu cytoplasmic domain revealed that the first α-helix was structurally important for CD4 binding and degradation [70,71]. Although the binding of Vpu to CD4 is necessary to induce CD4 degradation, it is not sufficient. Phosphorylation-deficient mutants of Vpu were shown to be unable to induce CD4 degradation while interacting with CD4 as efficiently as their WT counterpart [26,63,68,72,73]. A major finding in the mechanism underlying Vpu-mediated CD4 degradation was the discovery that phosphorylated Vpu proteins interacted with β-TrCP-1 [73] and β-TrCP-2 [74], two paralogous F-box adaptor proteins that are part of the cytosolic Skp1-Cullin1-F-Box (SCF) E3 ubiquitin (Ub) ligase complex [73]. β-TrCP functions as a substrate specificity receptor for the SCF^β-TrCP ^E3 Ub ligase and recognizes substrates, such as Vpu, upon phosphorylation of the two serine residues present within a conserved DS^P^GΦXS^P ^β-TrCP recognition motif [75] (Figure 1B). By directly interacting with the WD-repeat β-propeller of β-TrCP, Vpu is able to form a CD4-Vpu-β-TrCP ternary complex and as such brings CD4 and the other components of the E3 Ub ligase in close proximity so that *trans*-ubiquitination of CD4 could occur (Figure 2). In fact, biochemical and functional evidence in human cells as well as in yeast *S. cerevisiae *expressing Vpu and CD4 revealed that SCF^β-TrCP ^recruitment by Vpu results in polyubiquitination of the cytosolic tail of CD4 [76,77], thus marking the viral receptor for degradation by the cytosolic proteasome [37,78]. The function of Vpu in CD4 degradation is therefore very similar to that of E3 Ub ligase adaptors which link substrates to Ub ligases. Both β-TrCP1 and β-TrCP2 appear to be involved in the formation of a functional Vpu-SCF^β-TrCP ^E3 Ub ligase complex since small interfering (si) RNA silencing of both genes simultaneously was required to fully reverse Vpu-mediated CD4 degradation [74].

While the first helix of Vpu appears important for CD4 binding, the role of the second helix remains unclear. Deletion of the C-terminal 23 amino-acid residues or substitution of residues Val 64 to Met 70 abrogated Vpu-mediated CD4 degradation without, however, affecting CD4 binding [31,71]. A more recent study analyzed systematically the importance of each amino-acid within this region by alanine scan mutagenesis and identified Leu 63 and Val 68 as residues required for CD4 downregulation. Interestingly, in the case of Leu 63, substitution of this residue with Ala or Val, which maintain the predicted secondary structure of the helix, did not affect binding to CD4 or β-TrCP, but did abolish CD4 downregulation [70], suggesting that binding of Vpu to CD4 and recruitment of β-TrCP might not be sufficient to induce CD4 degradation and consequently that other process and/or interactions might be involved in this mechanism. It is interesting to note that the conserved Leu 63 and Val 68 are part of a conserved acidic/dileucine sorting signal, [D/E]XXXL[L/I/V] (Figure 1B), usually involved in the trafficking of membrane proteins between the endosomes and the TGN. The role of this sorting motif on Vpu exit from the ER as well as on its trafficking in general still remains undefined.

### ERAD-ication of CD4 by Vpu

The process of Vpu-mediated CD4 degradation is reminiscent of a cellular quality control process called ER-associated protein degradation (ERAD) that eliminates misfolded or unassembled proteins from the ER [79,80]. Abnormal proteins targeted by the ERAD pathway are usually recognized by a quality control system within the ER lumen and ultimately degraded by the cytoplasmic ubiquitin-proteasome system following transport across the ER membrane by a process called dislocation. However, unlike typical ERAD, which uses several membrane-bound E3 Ub ligases, including the HRD1-SEL1L complex [81], TEB4/MARCH-VI [82], and the GP78-RMA1 complex [83], Vpu-mediated CD4 degradation relies on the cytosolic SCF^β-TrCP ^E3 Ub ligase complex that is responsible for ubiquitination and degradation of non-ERAD substrates such as IκB [84] and β-catenin [85]. Consistent with these findings, genetic evidence in *S. cerevisiae *yeast expressing human CD4 and HIV-1 Vpu revealed that CD4 degradation induced by Vpu did not require HRD1 (E3), SEL1L and the E2 Ub conjugating enzyme UBC7, which are key components of the machinery responsible for ubiquitination of most ERAD substrates [76].

Recent studies have dissected in molecular terms the process of CD4 degradation mediated by Vpu and found that although the mechanism is distinct from typical ERAD it still shares similar features and, importantly, involves late stages components of the ERAD pathway. As a first step, Vpu was found to target CD4 for degradation by a process involving polyubiquitination of the CD4 cytosolic tail by SCF^β-TrCP ^[76,77] (Figure 2). Interestingly, replacement of cytosolic Ub acceptor lysine residues reduced but did not abolish Vpu-mediated CD4 ubiquitination and degradation, raising the possibility that the CD4 degradation induced by Vpu is not entirely dependent on the ubiquitination of cytosolic lysines [77]. Indeed, recent evidence revealed that more profound inhibition of degradation could be achieved by mutation of all lysine, serine and threonine residues in the CD4 cytosolic tail ([86] and our unpublished results). The ubiquitination process involved in Vpu-mediated CD4 degradation, therefore, resembles that involved in MHC-I downregulation induced by the mouse gamma herpesvirus (Gamma-HSV) mK3 E3 Ub ligase, which mediates ubiquitination of nascent MHC-I heavy chain (HC) cytosolic tail via serine, threonine or lysine residues to target MHC-I heavy chain for degradation by ERAD [87]. As a second step, although Vpu uses a non-ERAD E3 Ub ligase to induce CD4 degradation, it is co-opting downstream components of the ERAD pathway. In fact, the VCP-UFD1L-NPL4 complex, a key component of the ERAD dislocation machinery [88,89], was shown to be involved in CD4 degradation by Vpu (Figure 2). Using siRNA, a recent study reported a requirement of the valosin-containing protein (VCP) AAA ATPase p97 and its associated co-factors UFD1L and NPL4 in Vpu-mediated CD4 degradation [86]. Furthermore, the fact that mutants of p97 that are unable to bind ATP or to catalyze ATP hydrolysis exerted a potent dominant negative (DN) effect on Vpu-mediated CD4 degradation indicated that the ATPase activity of p97 was required for this process [77,86]. Further dissection of the role of the UFD1L and NPL4 cofactors in Vpu-mediated CD4 degradation revealed that while p97 appears to energize the dislocation process through ATP binding and hydolysis, UFD1L binds ubiquitinated CD4 through recognition of K-48 chains and NPL4 stabilizes the complex [86]. A role of the VCP-UFD1L-NPL4 complex in mediating the extraction of CD4 from the ER membrane, as observed for ERAD substrates [76] is consistent with data showing that Vpu was promoting the dislocation of ubiquitinated CD4 intermediates across the ER membrane [76,77]. Vpu, therefore, appears to bypass the early stages of ERAD including substrate recognition and ubiquitination by ERAD machinery components, but joins in the later stages beginning with dislocation by the VCP-UFD1L-NPL4 complex (Figure 2).

### Retention of CD4 molecules in the ER by Vpu?

Besides its role in CD4 ubiquitination and dislocation across the ER membrane so that receptor molecules be accessible to the cytosolic proteasome, a recent study provided evidence that Vpu plays also a role in the retention of CD4 in the ER [86]. It was initially found that Vpu was targeting CD4 molecules that were retained in the ER through formation of a complex with Env [62]. However, Magadan and colleagues found that even in absence of Env, large amounts of CD4 are retained in the ER in the presence of Vpu when ERAD is blocked [86]. Unexpectedly, these authors further showed that this retention was independent of the only interaction of Vpu and CD4 reported to date, which involves the cytosolic domain of both proteins. Rather, CD4 ER retention appeared primarily dependent on direct or indirect interactions involving the TM domains of both CD4 and Vpu. Indeed, a Vpu mutant containing a heterologous TM domain from the G glycoprotein of vesicular stomatitis virus (VSV) failed to retain CD4 in the ER. Although these results support a role of TM domain interactions in the retention of CD4 in the ER by Vpu, this interaction does not appear to rely on Vpu TM primary sequences since a Vpu mutant containing a scrambled TM domain was still competent at binding CD4 and at mediating CD4 degradation [69]. Vpu-mediated ubiquitination appears also to contribute to CD4 retention in the ER, but the mechanism remains unclear. Therefore, it appears that Vpu retains CD4 in the ER by the additive effects of two distinct mechanisms: assignment of ER residency through the TM domain and ubiquitination of the cytosolic tail. The findings of Magadan and colleagues supporting a role of Vpu in the retention of CD4 in the ER are not entirely consistent with those of a previous report, which showed that CD4 can efficiently traffic to the Golgi complex in presence of Vpu when CD4 is not retained in the ER by Env [36]. Indeed, using a subviral construct expressing Vpu and a mutant of gp160 defective for CD4 binding, Willey and colleagues found that despite the presence of Vpu the majority of CD4 acquired Endo H-resistant complex carbohydrates in the Golgi apparatus within 60 minutes after synthesis. Whether this discrepancy results from a difference in Vpu expression levels (Willey et al. expressed Vpu from a subviral vector while Magadan et al. used a codon-optimized Vpu construct that expresses much higher levels of the protein) or from differences in the assays used (prevention of CD4 degradation by blocking ERAD vs. allowing trafficking of CD4 by not blocking the receptor exit from the ER with Env) remains unclear. Clearly, more studies will be required to fully understand the mechanism through which Vpu confers on CD4 an intrinsic propensity to reside in the ER. Importantly, it will be critical to assess its relevance and contribution in the context of HIV infection where large amounts of CD4 are already complexed to Env gp160 in the ER.

Overall, based on previous findings and more recent evidence, a model of Vpu-mediated CD4 degradation emerges whereby Vpu might exert two distinct separable activities in the process of downregulating CD4: retention in the ER followed by targeting to a variant ERAD pathway (Figure 2).

## Role of Vpu in HIV-1 release and transmission

### Vpu promotes efficient release of HIV-1 particles in a cell-type specific manner

In addition to its effect on CD4 catabolism, Vpu was reported to promote the efficient release of virus particles from HIV-1-infected cells [7,38]. This finding was supported by electron microscopy (EM) studies, which revealed an accumulation of mature virions still tethered to the plasma membrane of infected T cells in the absence of Vpu [28,90]. Early studies demonstrated that the need of Vpu for efficient HIV-1 particle release was only observed in certain cell types. Notably, while Vpu-deficient HIV-1 release was drastically reduced in HeLa cells, monocyte-derived macrophages, and to a smaller extent in primary CD4+ T cells, normal viral particle release was observed in HEK293T, COS, CV-1, and Vero cells [39,91,92]. Importantly, the fact that Vpu could significantly enhance viral particle production by Gag proteins from HIV-2 or retroviruses distantly related to HIV-1, such as Visna and murine leukemia virus (MLV), suggested that the effect of Vpu was unlikely to require highly specific interactions with Gag proteins, but rather was more consistent with a model where Vpu enhanced retroviral release indirectly through modification of the cellular environment [40].

### Tetherin, the last obstacle to enveloped virus release

The notion that a cellular inhibitor of HIV-1 particle release antagonized by Vpu could be responsible for the inefficient release of Vpu-deficient HIV-1 in restrictive cells was suggested by the observation that heterokaryons between restrictive HeLa and permissive COS cells exhibited a restrictive phenotype similar to that displayed by HeLa cells [93]. Importantly, the fact that virions retained at the cell surface could be released by protease treatment suggested that a protein expressed at the cell surface was involved in the "tethering" of virions to the cell surface as opposed to a budding defect that prevented membrane separation [94]. Interestingly, cell types that allowed efficient release of Vpu-deficient HIV-1 viruses could become restrictive for viral release after type 1 IFN treatment, thus suggesting that the putative cellular protein that efficiently tethered virions on host cell surface was induced by type I IFN [95].

Almost simultaneously, the Bieniasz and Guatelli groups identified Tetherin as the cellular factor responsible for the inhibition of HIV-1 particle release and counteracted by the Vpu accessory protein [41,42]. Both groups found Tetherin to be constitutively expressed in cell lines that required Vpu for efficient particle release, like HeLa cells, but not in permissive HEK293T and HT1080 cells. Likewise, expression of Tetherin and its associated restrictive phenotype could be induced by IFN-α in permissive HEK293T and HT1080 cells and enhanced in Jurkat and primary CD4+ T cells. Furthermore, while introduction of Tetherin into HEK293T and HT1080 cells inhibited HIV-1 particle release in absence of Vpu, siRNA-directed depletion of Tetherin in HeLa cells led to efficient release of Vpu-deficient HIV-1 particles [41,42]. In addition to HIV-1, Tetherin has been shown to exert its antiviral activity against a broad range of enveloped viruses, including many retroviruses (alpharetrovirus, betaretrovirus, deltaretrovirus, lentivirus, and spumaretrovirus), filoviruses (Ebola and Marburg viruses), arenaviruses (Lassa virus), paramyxoviruses (Nipah virus) as well as Kaposi Sarcoma Herpes Virus (KSHV) [41,42,94,96-99], thus indicating that the process of restriction is unlikely to involve specific interactions with virion protein components.

### Tetherin: expression, structure and trafficking

Tetherin is a protein highly expressed in plasmacytoid dendritic cells (pDCs), the major producers of type I IFN, and in some cancer cells, while lower basal levels of expression are detected in bone marrow stromal cells, terminally differentiated B cells, macrophages, and T cells [100-104]. Its expression is strongly induced by type I IFNs in virtually all cell types [99,101,105-107], indicating that it is likely part of the innate defense response to virus infections.

**Figure 3:**
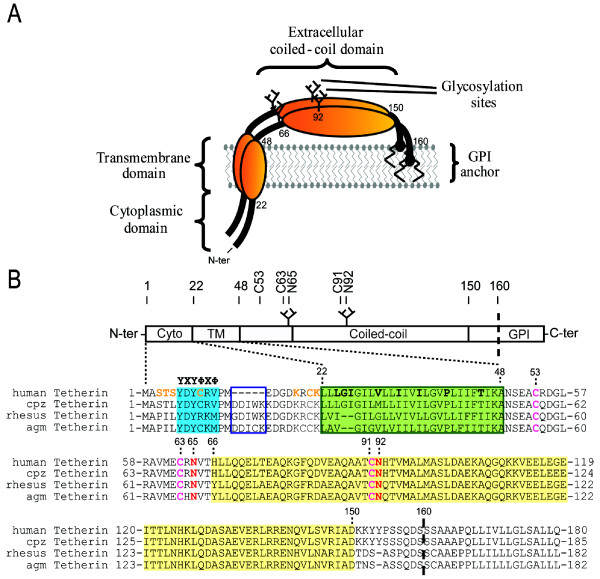
**Schematic representations of Tetherin**. Secondary and tertiary model of human Tetherin. Glycosylation sites at position 65 and 92 are shown as well as the GPI-anchor and the cytoplasmic, transmembrane (TM) and extracellular coiled-coil domains. The functional parallel dimeric state is shown here. (B) Tetherin topology. An amino-acid sequence alignment of human, chimpanzee, rhesus and African green monkey (agm) Tetherin alleles is shown below. Hyphens and bold letters represent respectively deletions and residues in human Tetherin under positive selection. Putative Ub-acceptor residues, cysteine residues involved in dimerization as well as N-glycosylation sites are labelled in orange, pink and red, respectively. Putative trafficking signals, the predicted transmembrane domain and the coiled-coil domain are highlighted in blue, green and yellow. The sites of interaction mapped for SIV Nef and HIV-1 Vpu are boxed in dark blue and dark green, respectively. Note that the SIV Nef-interacting region is deleted in human Tetherin. The site of cleavage prior to addition of the GPI lipid anchor is represented by the dashed line.

Tetherin is a glycosylated type II integral membrane protein of between 28 and 36 kDa with an unusual topology in that it harbors two completely different types of membrane anchor at the N- and C-terminus. It is composed of a short N-terminal cytoplasmic tail linked to a TM anchor that is predicted to be a single α-helix, a central extracellular domain predicted to form a coiled-coil structural motif, and a putative C-terminal glycophosphatidyl-inositol (GPI)-linked lipid anchor [105,108,109] (Figure 3A). This rather atypical topology is only observed in one isoform of the prion protein [110]. As a GPI-anchored protein, Tetherin is found within the cholesterol-enriched lipid domains from which HIV-1 and other enveloped viruses preferentially assemble and bud [108,111-113]. The protein is localized not only at the plasma membrane but also within several endosomal membrane compartments, including the TGN as well as early and recycling endosomes [29,108,112,114]. Clathrin-mediated internalization of human Tetherin is dependent upon a non-canonical tyrosine-based motif present in the cytoplasmic tail of the protein (Figure 3B), which appears recognized by α-adaptin but not the μ2-subunit of the AP-2 complex as it was initially reported for the rat Tetherin [109,112]. Moreover, after endocytosis, Tetherin delivered to early endosomes is subsequently transported to the TGN through recognition of the cytoplasmic domain by the μ1-subunit of the AP-1 complex [109], suggesting the involvement of the sequential action of AP-2 and AP-1 complexes in internalization and delivery back of Tetherin to the TGN. Although the current data is consistent with a model whereby Tetherin continually cycles between the plasma membrane and the TGN with a fraction targeted for degradation, it still remains to be determined whether the protein is indeed recycling from the TGN to the cell surface. Interestingly, recent findings from Rollason and colleagues revealed that Tetherin localizes at the apical surface of polarized epithelial cells, where it interacts indirectly with the underlying actin cytoskeleton, thus providing a physical link between lipid rafts and the apical actin network in these cells [111]. Whether this property of Tetherin relates to its activity as an inhibitor of HIV-1 release remains unknown.

The Tetherin ectodomain contains two N-linked glycosylation sites, three cysteine residues and a coiled-coil motif that mediates homodimerization (Figure 3B). Tetherin glycosylation was shown to be important for proper transport and perhaps folding of the protein [115]; but, however, appeared to be dispensable for its activity as a restriction factor [97,115,116]. In contrast, the presence of cysteine residues in the extracellular domain of Tetherin was found required for the anti-HIV-1 function of the protein, as mutation of all three cysteine residues to alanine abrogated the antiviral activity without affecting Tetherin's expression at the cell surface [115,116]. In that regard, it has been proposed that Tetherin forms a parallel dimeric coiled-coil that is stabilized by C53-C53, C63-C63 and C91-C91 disulfide bonds. Interactions within the coiled-coil domain and at least one disulfide bond formation are required for dimer stability and antiviral function [115,116]. Recently, a partial structure of the extracellular domain of Tetherin has been solved by X-ray crystallography by three different groups [117-119]. All these studies support a model in which the primary functional state of Tetherin is a parallel dimeric disulfide-bound coiled-coil that displays flexibility at the N-terminus.

### Tetherin directly cross-links HIV-1 virions on infected cell surface

Accumulating evidence suggest that Tetherin prevents viral release by directly cross-linking virions to host cell membranes. Additionally, restricted mature virus particles can also be found within intracellular endosomal structures [94], suggesting that following retention at the plasma membrane, tethered particles could be internalized and perhaps targeted for degradation in late endosomal compartments [120]. Consistent with a direct tethering mechanism, immuno-EM studies revealed that Tetherin is detected in the physical bridge between nascent virions and the plasma membrane as well as between virions tethered to each other [121,122]. In fact, both biochemical and immuno-EM evidence indicate that Tetherin is incorporated into virions [114,115,121,122]. Importantly, the view that Tetherin itself, without the need of any specific cellular cofactor, is responsible for tethering virions on host cell surface, is supported by evidence from the Bieniasz group. They elegantly showed that protein configuration rather than primary sequences is critical for the tethering phenotype. Indeed, an entirely artificial Tetherin-like protein consisting of structurally similar domains from three unrelated proteins (TM from transferrin receptor, coiled-coil from distrophia myotonica protein kinase and GPI anchor from urokinase plasminogen activator receptor), inhibited the release of HIV-1 and Ebola virus-like-particles in a manner strikingly similar to Tetherin [115].

**Figure 4:**
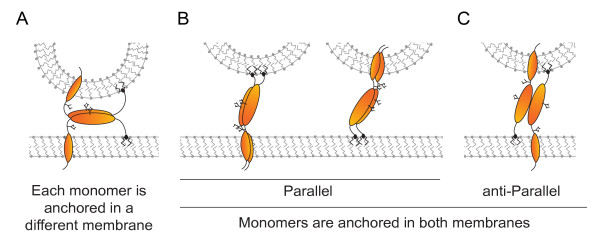
**Schematic representations of possible direct tethering modes**. (A) Tethering by interaction via the ectodomains of Tetherin dimers. One Tetherin molecule is inserted into the virus while the other is anchored into the cellular membrane. (B) Tethering by incorporation of one of the molecule anchors in the virus and the other in the cellular membrane. Different options are shown, including GPI anchors or transmembrane domains of parallel Tetherin homodimers incorporated into a virion and (C) both type of anchors from an antiparallel tetherin homodimer incorporated into virion. The fact that deleting either the GPI anchor or the TM domain prevents the restriction suggests that either configuration A and C are not important contributors of the tethering process or that a single tethering domain is not sufficient to retain virions at the cell surface. Indeed, it is also conceivable that all these potential configurations may contribute to the restrictive activity albeit to different extent.

Although strong evidence for a direct tethering mechanism exists, the precise topology of the Tetherin dimers and the definition of the molecular interfaces retaining nascent virions at the cell surface remain open questions. For instance, it is not clear whether both membrane anchors remain in a single membrane surface and virions are retained by interaction between two Tetherin ectodomains (Figure 4A) or, if both Tetherin anchors can be incorporated in different membrane surfaces (Figure 4B). The fact that removal of either the cytoplasmic tail or the GPI anchor abrogates the antiviral activity of Tetherin [41,115] supports a model whereby Tetherin is a parallel homodimer with one set of anchors in the host membrane and the other in the virion membrane (Figure 4B). This data also suggest that anti-parallel dimers with monomeric links to membranes (Figure 4C) do not exist or cannot effectively tether. It remains unclear whether the parallel dimers of Tetherin that span the plasma and viral membranes have a preference for which membrane anchor ends up in the cell membrane or in the virion although there is some evidence that the TM anchor is favored in the virus membrane and the TM anchor in the cell [115]. While this membrane spanning model is consistent with the structural properties of Tetherin, there are, however, two caveats to this model. First, treatment of tethered virions with the GPI anchor-cleaving enzyme, phosphatidyl inositol-specific phospholipase C (Pi-PLC), did not effectively release virions from the cell surface [122]. Second, based on the structural data, the maximum distance that can be bridged by Tetherin in the configuration outlined in Figure 4 is about 17 nm [117-119]. However, the distance between the plasma membrane and tethered viral membranes observed in EM studies is frequently significantly larger than that distance [121,122]. One alternative model to the membrane spanning domain model is that. individual Tetherin monomers are anchored at both end to the viral or plasma membranes but associates with each other through dimers or higher order structures (Figure 4A). Although this model explains a requirement for dimerization, it does not explain why Tetherin requires both of its membrane anchor domains for its antiviral activity [41,115]. Moreover, in this model, Tetherin would tether virus particles quite close to the plasma membrane (3-5 nm), a distance not supported by EM analysis [41,115,121,122]. Clearly more studies are required to fully understand how Tetherin dimers tether newly formed virions to host cell surface.

### Effect of Tetherin on HIV-1 cell-to-cell transmission

Increasing evidence suggests that HIV-1 can spread directly between T cells by forming a polarized supra-molecular structure termed a virological synapse, whereby nascent virions are recruited to intimate adhesive contacts between infected and uninfected cells [55,56]. Since Vpu-deficient HIV-1 particles accumulate at the cell surface as a result of Tetherin-mediated restriction, it is unclear whether these restricted virions can undergo cell-to-cell transfer or whether Tetherin restricts spread via virological synapses in addition to inhibiting the release of cell-free virions. Tetherin was recently reported to inhibit productive cell-to-cell transmission from Tetherin-positive donor cells (HEK 293T, HeLa and T-cells) to target lymphocytes without preventing the formation of virological synapses [123]. Interestingly, in the presence of Tetherin, Vpu-deficient viruses accumulated at the synapses and were essentially transferred to target cells as large abnormal aggregates. These viral aggregates were found to be impaired in their ability to fuse to target cells and as such did not efficiently promote productive infection after transfer. These findings contrast with results recently reported by Jolly and colleagues which showed that Tetherin does not restrict virological synapse-mediated T-cell to T-cell transfer of Vpu-deficient HIV-1 [124]. In fact, this study showed that in some circumstances Tetherin might promote cell-to-cell transfer either by mediating the accumulation of virions at the cell surface or by regulating the integrity of the virological synapse. These latter findings are consistent with a previous study that reported that *in vitro *selection of HIV-1 to spread via cell-to-cell contact in T-cell lines led to the emergence of viral variants with mutations in both the Env and the Vpu proteins [125]. Likewise, earlier observations showed that WT and Vpu-deficient HIV-1 production usually peak at the same time during a spreading infection even though less Vpu-deficient virus is released in the extracellular milieu [28,38]. The contrasting results obtained by Jolly and colleagues [124] and Casartelli and colleagues [123] might reflect cell type dependent variations in the levels of Tetherin expression since the two studies used distinct Tetherin-expressing cell donor systems. It is indeed possible that under high Tetherin expression conditions such as those prevailing in macrophages and dendritic cells (DCs), HIV-1 cell-to-cell transmission might be impaired, whereas at lower levels, such as in T-lymphocytes, Tetherin may not restrict or may even contribute to cell-to-cell transmission. In that regard, Schindler and colleagues recently showed that a Vpu mutant (S52A) that displayed an impaired Tetherin antagonism was unable to replicate efficiently in macrophages, while it spread as well as the wild type virus in *ex vivo *lymphoid tissue (HLT) or peripheral blood lymphocytes [103]. Therefore, one role of Vpu would be to maintain a balance between cell-free and cell-to-cell HIV-1 spread in the face of antiviral immune responses.

### Potential roles of Tetherin in innate immunity

HIV-1 infection induces pDCs to produce a broad range of type I IFN through the activation of Toll-like receptors 7 and 9 (TLR7 and TLR9) [126,127]. Type I IFN activates natural killer (NK) cells, myeloid DCs, T cells, B cells, and macrophages and induces expression of several hundreds of different IFN-stimulated genes, including Tetherin. In turn, it was recently reported that human Tetherin is the natural ligand for ILT7, a protein that is expressed exclusively on pDCs [107]. Binding of Tetherin to ILT7 was found to trigger a signaling pathway that negatively modulates TLR7- or TLR9-mediated type I IFN and proinflammatory cytokine secretion, thus establishing a negative feedback loop in which IFN-induced Tetherin binding to the ILT7-FcεRIγ complex signals the inhibition of additional IFN production [107]. Therefore, in addition to its anti-viral function, Tetherin might also have a role in modulating pDC's IFN responses as well as inflammatory responses to virus infection. Whether or not Vpu interferes with this Tetherin immunomodulatory function during HIV-1 infection remains an open question.

### HIV-1 riposte to Tetherin-mediated restriction

How Vpu counteracts the antiviral activity of Tetherin has attracted considerable attention since the discovery of the restriction factor. A key observation made early on was that Vpu downregulated Tetherin from the cell-surface [42]. This reduction of Tetherin levels at the cell surface correlated with the enhancement of HIV-1 particle release observed upon Vpu expression. Since Tetherin restricts HIV-1 virus particle release at the plasma membrane, removal of Tetherin from its site of tethering action represents an intuitive model through which Vpu could counteract this cellular restriction, although this model has been challenged [102]. Reduction of Tetherin at the cell-surface is likely to prevent cross-linking of cellular and viral membranes, which implies that virions released from Vpu-expressing cells would be devoid of Tetherin molecules. Although this notion is supported by the decreased co-localization between Tetherin and Gag in presence of Vpu [41,96,128], biochemical analyses revealed that Vpu expression decreases only partially Tetherin accumulation in released virus particles [115,121,122]. Furthermore, immuno-EM studies showed that virions produced from Vpu-expressing cells still incorporate Tetherin albeit at a lower density [114,122]. These results suggest that either a threshold level of virion-associated Tetherin is required to mediate the restriction or alternatively removal of Tetherin from specific plasma membrane microdomains could underlie the mechanism by which Vpu antagonizes Tetherin. In that regard, Habermann and colleagues reported that Vpu would be more efficient at downregulating Tetherin outside HIV-1 assembly sites [114], thus reducing the ability of the plasma membrane to retain fully released Tetherin-containing virions. Interestingly, a recent study provided evidence that partitioning of Vpu in lipid rafts would be required to promote virus particle release [129]. It will be important to determine whether Vpu targets a pool of Tetherin located in specific microdomains of the plasma membrane or whether the viral antagonist targets the restriction factor independently of its distribution at the plasma membrane.

### Vpu, a versatile Tetherin antagonist

Several mechanisms have been proposed to explain how Vpu can downregulate Tetherin from the cell surface and as a result antagonize its antiviral activity on HIV-1 release. These include proteasomal or endo-lysosomal degradation of the restriction factor and/or alteration of its trafficking toward the cell surface, resulting in intracellular sequestration. Although mechanistically distinct, these modes of antagonism all rely on the ability of Vpu to bind Tetherin since the restriction imposed by Tetherin on viral particle release can be restored by mutations disrupting their mutual association [130-134]. Such mutations have been all mapped so far to either Vpu or Tetherin TM domains, thus strongly suggesting that the two proteins associate through their respective TM regions [131,132,135-137]. Evidence for each of the proposed Vpu anti-Tetherin mechanisms are reviewed and discussed below.

#### i) Vpu-mediated degradation of Tetherin

Vpu expression was found to decrease the total steady-state levels of Tetherin [128,130,134,138,139]. This depletion occurs at a post-transcriptional step since levels of Tetherin transcript are not affected by Vpu [130,134]. Importantly, pulse-chase experiments revealed that Vpu accelerates the turnover of endogenous Tetherin [130,131,139]. As observed with Vpu-mediated CD4 degradation, recruitment of β-TrCP was found to be required for Vpu-mediated Tetherin degradation since phosphorylation-deficient Vpu mutants did not alter Tetherin turnover [130,131]. Likewise, siRNA-mediated depletion of β-TrCP or inactivation of the SCF^β-TrCP ^E3 Ub ligase by overexpression of a DN mutant of β-TrCP, β-TrCPΔF, which binds Vpu but is unable to link it to the SCF^β-TrCP ^E3 ligase complex, abolished Vpu-mediated Tetherin degradation, indicating that recruitment of the SCF^β-TrCP ^complex is critical for Vpu-mediated Tetherin degradation [128,130,133,134]. In that regard, siRNA depletion experiments revealed that Vpu takes specifically advantage of the cytoplasmic β-TrCP-2 isoform, but not the nuclear β-TrCP-1, to achieve Tetherin degradation [128,130,133]. Finally, complementing this set of functional evidence, β-TrCP was found in a ternary complex together with Vpu and Tetherin [133,134], but was not critical for the association of Vpu to Tetherin [130].

This degradative process was initially thought to be proteasomal in nature since long treatment with proteasomal inhibitors prevented exogenously-expressed Tetherin degradation in presence of Vpu in HEK 293T cells [134,138-140]. Furthermore, overexpression of Ub K48R, a DN mutant of Ub, which interferes with polyubiquitination, prevented Tetherin degradation [134]. Consistently, Vpu was found to promote the β-TrCP-mediated ubiquitination of Tetherin cytoplasmic tail on serine, threonine, lysine and cysteine residues at least in HEK293T [141] (Figure 3B). Interestingly, depletion of the ERAD component, AAA ATPase p97, affected Vpu-mediated Tetherin degradation [134], suggesting that Vpu could mediate Tetherin proteasomal degradation in the ER through an ERAD-like process. However, there are several caveats with the data supporting proteosomal degradation. First, the results were often obtained by overexpression of epitope tagged-Tetherin in non restrictive HEK 293T cells, an experimental setting which results in the accumulation of immature Tetherin molecule within the endoplasmic reticulum [116]. Second, long exposure with proteasome inhibitors or Ub K48R overexpression are not always an unambiguous evidence of ubiquitin-proteasome degradation since these processes can deplete free Ub, thus affecting indirectly Ub-dependent trafficking and/or lysosomal degradation [142,143]. Accordingly, inhibitors of lysosomal sorting and acidification, such as bafilomycin A and concanamycin were found to inhibit Vpu-mediated Tetherin degradation and could interfere with Tetherin downregulation from the cell-surface [128,130]. This type of degradation is indeed consistent with the recent observation that Tetherin undergoes monoubiquitination on cytoplasmic lysines 18 and/or 21 in presence of Vpu [144]. Taken together, these findings support a model whereby Tetherin undergoes degradation in lysosomes in presence of Vpu. However, proteasomal degradation cannot be completely excluded, at this point.

#### ii) Vpu-mediated intracellular sequestration of Tetherin

**Figure 5:**
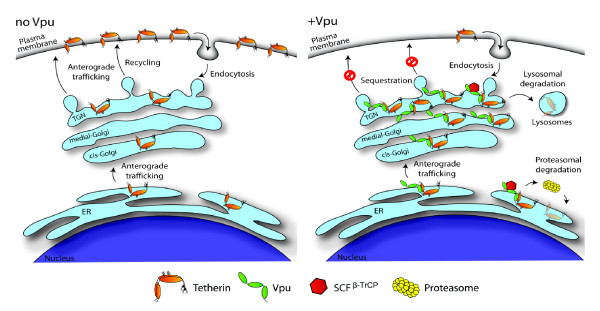
**Unified model of Vpu-mediated cell-surface Tetherin downregulation**. Tetherin traffics along the anterograde trafficking pathway and reaches the plasma membrane. The protein is endocytosed in clathrin-coated pits, transported to the TGN and most probably recycles back to the cell surface. Upon expression of Vpu, Tetherin is forming complexes with the viral protein, thus trapping the restriction factor in the TGN, away from sites of viral assembly at the plasma membrane where Tetherin is cross-linking progeny virions. Vpu could intercept endocytosed Tetherin as well as Tetherin arriving from the ER although this remains to be determined. Subsequently, Vpu could induce Tetherin ubiquitination through recruitment of β-TrCP-2, leading to a stronger retention in the TGN. Sequestered ubiquitinated Tetherin conjugates could ultimately be targeted for proteosomal and/or lysosomal degradation. As such, Vpu-mediated Tetherin degradation may represent a complementary mechanism that Vpu could exploit to reach optimal Tetherin antagonism, perhaps, in specific cellular environments.

Although Vpu expression induces Tetherin degradation in most cellular systems studied to date, several lines of evidence suggest that degradation of Tetherin *per se *cannot entirely account for Vpu-mediated Tetherin antagonism. For instance, Vpu was found to decrease total cellular Tetherin to a lesser extent than cell-surface Tetherin in HeLa cells [128]. Furthermore, Vpu mutants that contain mutations in the DS^P^GΦXS^P ^β-TrCP recognition motif that render them deficient for directing β-TrCP-dependent degradation of Tetherin are still able to partially [39,42,128,130,134] or in some instances to totally [24,102,103] overcome the Tetherin-mediated particle release restriction. Moreover, Vpu-mediated Tetherin degradation is a relatively slow process [130,131] (half-life of ~8 h is decreased by ~2-fold in presence of Vpu) as compared to the efficient CD4 receptor degradation induced by Vpu (half-life of ~6 h is decreased by ~25-fold in presence of Vpu [36]). There is now increasing evidence for the existence of an anti-Tetherin mechanism that is distinct from degradation of the restriction factor. In that regard, recent evidence showed that Vpu did not promote Tetherin endocytosis [128,131], but rather induced a re-localization of the antiviral factor to a perinuclear compartment that extensively overlapped with the TGN marker, TGN46, and Vpu itself, thus leading to a specific removal of cell-surface Tetherin [131,144,145]. These findings are indeed consistent with previous data showing that proper distribution of Vpu in the TGN is critical to overcome Tetherin restriction on HIV-1 release [29]. Overall, it appears that Vpu may antagonize Tetherin, at least in part, by sequestering the protein intracellularly through alteration of its normal anterograde trafficking. Importantly, mutations of the putative Ub-acceptor lysine residues, K18 and K21, in the cytosolic tail of Tetherin (Figure 3B), completely abolished Vpu-mediated monoubiquitination [144] and degradation [140] of the restriction factor without, however, affecting its antagonism by Vpu. These findings provide genetic evidence that Tetherin ubiquitination/degradation and Tetherin antagonism may be two separable Vpu activities. Moreover, since the lysine-less Tetherin mutant was still found to be downregulated at the cell surface in presence of Vpu, it appears that Tetherin downregulation and ubiquitination/degradation may not be as strictly linked during Vpu-mediated antagonism [140,144]. These unexpected results contrast, however, with previous data showing that Vpu mutants that are unable to recruit β-TrCP (and as such are predicted to be unable to mediate Tetherin ubiquitination/degradation), such as phosphorylation-deficient Vpu mutants, display an attenuated Tetherin antagonism (~50% of WT Vpu) [128,131,134,146]. Likewise, depletion of β-TrCP-2 or expression of a DN mutant of β-TrCP, β-TrCPΔF, could partially inhibit Vpu-mediated Tetherin antagonism [128,130,134]. The recent results of Tokarev and colleagues indicating that β-TrCP-mediated polyubiquitination of the Tetherin cytoplasmic tail specifically on serine and threonine residues is critical for Vpu-mediated Tetherin antagonism, while having little effect on the stability of the restriction factor in presence of Vpu, might provide some clues on the role β-TrCP on Tetherin antagonism. Based on these results, it is indeed possible that Vpu-mediated polyubiquitination of Tetherin via β-TrCP, might enhance the sequestration of the restriction factor without necessarily leading to Tetherin degradation [141]. Taken together, these recent findings suggest that Tetherin antagonism by Vpu precedes and may not be dependent on degradation of the restriction factor, but rather results in the sequestration of Tetherin away from budding virions (Figure 5). Clearly more studies are needed to evaluate the contribution of Tetherin ubiquitination and degradation in the mechanism through which Vpu antagonizes the restriction on HIV-1 particle release especially in cells that are natural target for infection.

Although current evidence is consistent with a model whereby antagonism of Tetherin by Vpu involves sequestration of the restriction factor in a perinuclear compartment, it is unclear whether Vpu subverts recycling and/or other intracellular sorting steps of Tetherin. Mutation of the overlapping Tyrosine (Tyr6 and Tyr8) trafficking signals in the Tetherin cytoplasmic tail (Figure 3B), which blocks the protein natural pathway of endocytosis, did not abolish the sensitivity to Vpu in non-restrictive cells (HEK 293T, HT1080) transiently [131,133,145] or stably expressing the Tetherin mutant [140]. Thus, it appears that Tetherin intracellular sequestration may occur before endocytosis of the restriction factor from the cell surface and as such involve newly synthesized Tetherin en route to the PM [131,133]. However, it cannot be ruled-out completely that Vpu could interact with endocytosed Tetherin and prevent its recycling back to the cell surface since previous data demonstrated a requirement for the recycling endosomes in Vpu function [30], and recent studies reported that AP2 depletion [128] or over-expression of dominant DN mutant of Dynamin [133] could partially interfere with Vpu-mediated downregulation of Tetherin from the cell surface. Moreover, Tetherin is accumulated in the presence of Vpu in structures just beneath the plasma membrane that could correspond to early and/or recycling endosomes [114]. Indeed, since Vpu is produced from a Rev-regulated gene expressed late during the virus life cycle, the direct removal of Tetherin from the plasma membrane via endosomal trafficking may be critical to ensure a rapid and efficient neutralization of Tetherin's antiviral activity.

### Tetherin, a common enemy

While HIV-1 Vpu was the first anti-Tetherin factor discovered, there is now a growing list of virus-encoded proteins harboring anti-Tetherin activities. Namely, KSHV K5, HIV-2 Env, and Ebola gp were found to overcome human Tetherin antiviral activity apparently through distinct mechanisms [98,99,144,147-149] (Table 1). Nef from SIVcpz, SIVgor, SIVagm and SIVsmm, Env from SIVtan (SIV from Tantalus monkey) and Vpu from SIVgsn, SIVmon and SIVmus were also reported to antagonize Tetherin from their corresponding hosts [150-153]. Their strategies differ, though. The KSHV K5 protein, which is a membrane associated Ring-CH (MARCH) Ub ligase, induces a species-specific downregulation of human Tetherin from the cell surface by inducing ubiquitination of Tetherin cytoplasmic lysines and targeting the restriction factor to lysosomes for degradation [99,144]. In contrast, HIV-2 Env or SIVtan Env do not display any ability to induce Tetherin degradation [145,147-149]. Instead, these envelope proteins redistribute Tetherin from the plasma membrane to a perinuclear compartment that appears to correspond to the TGN most probably by a sequestration mechanism [145,148,149]. However, in contrast to Vpu, the determinants controlling Tetherin sensitivity to HIV-2 and SIVtan Env proteins are located in the restriction factor ectodomain [148,149]. Studies on HIV-2, SIVtan Env and KSHV K5 indicate that both sequestration and degradation represent potent mechanisms by which Tetherin antiviral activity can be overcomed. The fact that HIV-1 Vpu can exploit both mechanisms confirms its great versatility and might perhaps explain the efficient counteraction that it displays against human Tetherin. Although the precise mechanism underlying Tetherin antagonism by Nef remains to be determined, it was found that SIVmac Nef downregulates rhesus Tetherin from the cell surface and as shown for Vpu-mediated Tetherin antagonism, this downregulation correlated with enhanced SIVmac particle release [150,152]. Finally, the antagonism of human Tetherin by Ebola gp is still not entirely understood. The case of Ebola gp is particularly interesting since it is the only known anti-Tetherin factor that does not appear to downregulate Tetherin from the cell surface to promote the release of Ebola virus-like particle [147]. Even more surprising, interaction between Tetherin and Ebola gp would not require any specific sequence, a feature that is unique among Tetherin antagonists identified to date [147]. Therefore, it appears that Ebola virus gp might use a novel mechanism to neutralize Tetherin restriction. Clearly, comparative studies of these anti-Tetherin viral factors will not only further our understanding of their mechanisms of action but will also provide key information on host cell processes involved in Tetherin antagonism.

**Table 1:** Anti-Tetherin mechanisms reported for the known Tetherin antagonists

Tetherin antagonists	Binding	Mechanisms	References
HIV-1 Vpu	Yes	Proteosomal degradation	[134,138,139]
		Lysosomal degradation	[128,130]
		Sequestration in TGN and/or endosomes	[131,145]

HIV-2 Env	Yes	Intracellular sequestration	[145,148]

SIV Nef	?	Increased internalization from the cell-surface?	[150-152]

SIVtan Env	?	Intracellular sequestration	[149]

KSHV K5	?	Lysosomal degradation	[99,144]

Ebola gp	Yes	?	[98,147]

## Role of Vpu in primate lentivirus cross-species transmission and the emergence of pandemic HIV-1 strains

Genes encoding for restriction factor, such as TRIM5-α, APOBEC3G and Tetherin undergo rapid evolution, a process also known as positive selection, most likely as the result of the selective pressure imposed by new emerging viral pathogens or to escape from viral antagonists during the millions of years of virus-host co-evolution [154-156]. Consequently, these restriction factors display a high degree of sequence divergence and constitute potent limiting barriers to virus cross-species transmission because viral antagonists usually function in a species-specific manner. In fact, sequence alignment of Tetherin from different primate species reveals important selective genetic changes that lead to amino-acid substitution or deletion primarily in the cytoplasmic and TM domains of the protein, regions that are now known to be targeted by Tetherin antagonists [138,156,157] (Figure 3B).

**Figure 6:**
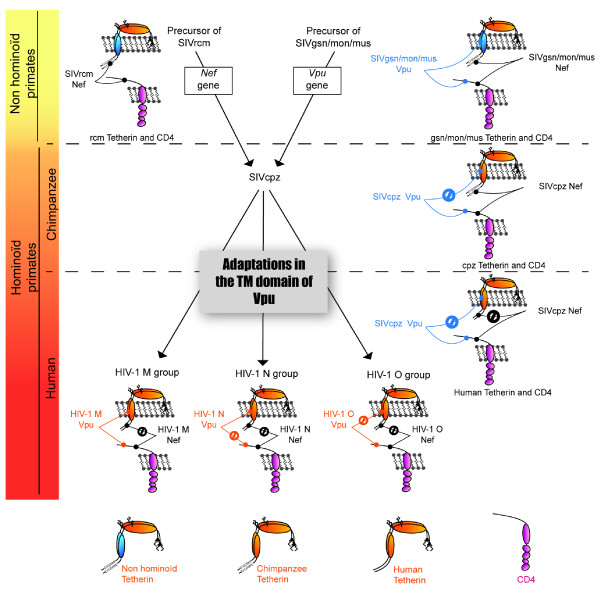
**Adaptations of primate lentiviruses during cross-species transmission and the emergence of pandemic HIV-1 strains**. The SIV from chimpanzee is believed to result from recombination events through successive cross-species transmission between the precursors of the SIVgsn/mon/mus and the SIVrcm lineages. The transmembrane domain of Tetherin evolved primarily during transition from the non hominoïd lineage to the hominoïd lineage, explaining why the Vpu protein inherited from the SIVgsn/mon/mus lineage does not exhibit any activity against chimpanzee Tetherin. After transmission from chimpanzees to humans, SIVcpz was unable anymore to use Nef to counteract Tetherin due to a deletion of five amino acids in the cytoplasmic domain of human Tetherin, which usually confers responsiveness to Nef. During evolution/adaptation from SIVcpz to HIV-1, modifications mapped to two regions of the Vpu transmembrane domain have conferred human Tetherin a susceptibility to Vpu, except in the case of the HIV-1 group O. Furthermore, the HIV-1 group N Vpu somehow has lost its ability to mediate CD4 degradation in the process. Only the pandemic HIV-1 group M harbors the two primary Vpu functions. Susceptibility of the transmembrane domain of Tetherin to Vpu is represented by similar colour pairing. The deletion of the five amino acids in human Tetherin cytoplamic tail is represented by the absence of the D/GDIWK sequence. The color gradient indicates co-evolution between SIV/HIV and host.

These genetic changes in Tetherin sequence have shaped the evolution of primate lentiviruses and influenced at least in part their ability to transmit across species. There is evidence suggesting that SIVcpz, the precursor of HIV-1, results from recombination events between the precursors of the SIVgsn/mon/mus and the SIV from red capped mangabeys (SIVrcm) lineages [158] (Figure 6). The precursor of SIVgsn/mon/mus that contributed to the 3'-half of the SIVcpz/HIV-1 genome, most likely harbored a *vpu *gene product able to both induce CD4 degradation and antagonize Tetherin since all descendants do. Since it is believed that the ancestor of the SIVrcm lineage used Nef to antagonize Tetherin, given that this lineage does not encode a *vpu *gene, the chimeric virus that gave rise to SIVcpz contained two potential Tetherin antagonists. However, the SIVcpz Vpu protein inherited from the SIVgsn/mon/mus lineage is devoid of any activity against the chimpanzee and human Tetherin [151,153,157].

The resistance displayed by the chimpanzee Tetherin to the Vpu protein originating from the SIVgsn/mon/mus lineage results from the high sequence variability in the TM domain of Tetherin reported between non-hominoid primates (monkeys such as rhesus or African green monkey) and hominoid primates (such as chimpanzees, gorillas and humans) [151,156,157] (Figure 1B). Indeed, this TM domain was demonstrated to be the site conferring Vpu susceptibility and binding [131,132,136,138,156]. As a result Nef, and not Vpu, evolved to become an effective Tetherin antagonist in SIVcpz-infected chimpanzee, most likely because the cytoplasmic domain sequence DDIWK targeted by Nef is somewhat less divergent between chimpanzee and monkey than the TM domain targeted by Vpu [150-153,157]. The deletion of sequences, highly subjected to adaptation and recognized by Nef, in human Tetherin is hypothesized to be the result of a previous encounter with a viral pathogen during human evolution that used a Nef-like protein, if not Nef itself, to antagonize Tetherin [157]. Conversely, few differences were observed in the TM domain of Tetherin between chimpanzee and human Tetherin, suggesting that Vpu has not driven Tetherin adaptation for a long period in primate evolution and accounting for the ability of the HIV-1 Vpu to counteract the chimpanzee Tetherin [157] (Figure 3B).

The inability to use Nef as a human Tetherin antagonist following SIVcpz cross-species transmission to human has likely led SIVcpz to proceed to a ''neofunctionalization'' of its initially incompetent Vpu protein in order to efficiently overcome the restriction imposed by human Tetherin (Figure 6). In support of this adaptation mechanism, it was recently reported that the differential ability of HIV-1 and SIVcpz Vpu to antagonize human Tetherin could be mapped to two regions of the TM domain of Vpu (amino-acid residues 1-8 and 14-22). Importantly, SIVcpz Vpu was completely able to overcome the human Tetherin restriction when these two regions were substituted for those from HIV-1 Vpu [157]. Furthermore, analysis of a SIVcpz strain (LB7) that represents the closest relative to HIV-1 group M revealed that the LB7 Vpu allele is predicted to need 7 minimal adaptations within these two critical regions of the TM domain to gain the ability to antagonize human Tetherin [157]. Consistent with these findings, mutagenesis of the prototypical NL4.3 Vpu TM domain identified three amino acid positions A14, W22 and, to a lesser extent A18, as Vpu residues critical for Tetherin binding and antagonism [135] (Figure 1B). Interestingly, these residues are predicted to align on the same face of the Vpu TM α-helix and as such might potentially be part of the interface that directly or indirectly interacts with the TM domain of human Tetherin.

The adaptation towards Vpu specifically shaped to counteract the hominoid Tetherin is at the center of the lineage-specific anti-Tetherin activity harbored by HIV-1 Vpu [138,151,156]. In line with this observation, substituting the TM domain of the human Tetherin with that from African green monkey or rhesus monkey Tetherin abrogated the chimeric protein sensitivity and binding to HIV-1 Vpu [131,132,156]. Indeed, analysis of the residues within the TM domain of human Tetherin, which determines the susceptibility to HIV-1 Vpu-mediated antagonism, revealed that one single substitution mutation for a residue found in the monkey Tetherin (T45I) combined to a deletion of two amino acids (G25, I26), absent from agm Tetherin, resulted in an efficient restriction of wild-type HIV-1 [156]. More recently, using a live cell based assay to monitor interaction, Kobayashi and colleagues performed a detailed analysis of the amino-acid residues within the TM domain of human Tetherin that are involved in Vpu binding [136]. This study identified three amino acid residues (I34, L37 and L41) as critical determinants for Vpu interaction and susceptibility [136] (Figure 3B). Furthermore, consistent with previous studies [132,138,156,159], they found that the integrity of the _22_L-L-L-G-I_26 _amino acid sequence, which is indeed altered by a deletion of two amino acids in agm/rhesus Tetherins (deletion of L22, L23 or L24, G25 or G25, I26, depending on the alignment), and/or conservation of a threonine at position 45 is required for the antagonism by Vpu (Figure 3B). On the basis of computer-assisted structural modeling and mutagenesis data, this study proposes that alignment of amino-acids of I34, I37, L41 and T45 on the same helical face in the TM domain, a positioning apparently governed by the presence of L22 and L23 (or perhaps the integrity of the _22_L-L-L-G-I_26 _amino-acid sequence), is crucial for human Tetherin antagonism by Vpu. Intriguingly, while mutation at position T45 affected the sensitivity of human Tetherin to Vpu, it did not significantly affect the formation of a Tetherin-Vpu complex, suggesting that interaction of Vpu to Tetherin alone is not sufficient to mediate Tetherin antagonism. Further studies will be necessary to address this potentially important issue.

Phylogenetic analyses indicate that at least three independent cross-species transmissions of SIVcpz gave rise to HIV-1 group M (main), N (non-M, non-O) and O (outlier) [160]. The functional properties acquired by Vpu proteins during these three independent transmissions of SIVcpz to humans have been recently analyzed and shown to have perhaps determined the propensity of these different groups to spread efficiently [151]. The Vpu protein of the HIV-1 M group responsible for the global HIV-1 pandemic was found to efficiently antagonize human Tetherin and to induce CD4 degradation. In contrast, the non-pandemic HIV-1 group O Vpu was found to have preserved its ability to mediate CD4 degradation but displayed a very weak activity against human Tetherin, while the rare HIV-1 group N gained some anti-human Tetherin activity, but lost its ability to degrade CD4 (Figure 6). These observations have led Kirchhoff and colleagues to propose that the acquisition of a fully competent Vpu protein able to antagonize Tetherin and mediate CD4 degradation may have facilitated the global spread of the HIV-1 M group, as opposed to the N and O groups whose distribution has remained very limited and focused to West Africa [151]. Nevertheless, the fact that HIV-1 from the N and O groups are still able to cause AIDS in infected individuals suggests that Vpu-mediated CD4 degradation and Tetherin antagonism may not be biological activities required for HIV-1 dissemination within an infected individual but rather functions that are critical for efficient transmission between individuals. A similar parallel can also be established with HIV-2, whose geographical distribution has been limited in comparison to HIV-1 [161]. As discussed previously, HIV-2 antagonizes human Tetherin using its Env glycoprotein since its genome does not encode a Vpu protein and its Nef protein cannot counteract human Tetherin [145,148]. Interestingly, HIV-2 Env was found to antagonize Tetherin less efficiently than HIV-1 Vpu, perhaps, because its mode of action solely involves a trapping of the restriction factor in the TGN [148]. However, the fact that this virus uses a structural protein to both downregulate CD4 (through complex formation in the ER) and Tetherin, could have also entailed a greater fitness cost to the virus than the use of accessory proteins such as Vpu or Nef, thus explaining in part its attenuated virulence in comparison to HIV-1 [161]. Therefore, it will be interesting to assess in the future whether Tetherin antagonism and/or CD4 degradation promote human-to-human HIV-1 transmission, perhaps by increasing the secretion of infectious virions into the genital fluids.

## Conclusions and perspectives

As molecular and cellular details about the mechanisms through which Vpu mediates CD4 degradation and antagonizes Tetherin emerge, it is becoming increasingly clear that the acquisition of a multifunctional Vpu protein by HIV-1 has played a crucial role in the virulence of this virus. Although the role of Vpu as an antagonist of Tetherin has obvious implications on virus secretion and potentially on the transmission efficiency from human to human, its effect on the dissemination of HIV-1 following establishment of infection is less clear, especially since the potential restricting effect of Tetherin imposed on cell-to-cell viral transmission does not appear to significantly interfere with viral spread at least in T lymphocytes [124]. It is possible that once infection is established in lymphoid tissue, such as the gut-associated lymphoid tissue (GALT), a major site of HIV-1 replication, cell-to-cell transfer may be an important mode of HIV-1 dissemination as opposed to a transmission via cell-free virus. One might speculate that Tetherin antagonism by Vpu may be less critical at this point. More studies will be required in the future to address this issue and to assess whether enhanced cell-to-cell transmission might not be a way for HIV-1 to escape or even tolerate Tetherin restriction. The contribution of Vpu-mediated CD4 degradation to HIV-1 spread remains unclear and will need to be further addressed. As discussed above, previous studies have suggested that this function would facilitate virion assembly by releasing Env precursor gp160 trapped with newly synthesized CD4 in the ER and maintain the release of fully infectious virions (reviewed in [21]). In that regard, experiments using the simian-human immunodeficiency virus (SHIV) model in pigtail macaques, a host that does not express a Tetherin protein susceptible to HIV-1 Vpu, suggested that the ability of Vpu to downregulation CD4 expression directly correlated with the progression of disease in this animal model [162,163]. Macaques inoculated with SHIV expressing a Vpu protein with mutations of the two phosphoserines involved in the recruitment of β-TrCP did not show any evidence of CD4+ T cell depletion and maintained significantly lower viral loads than macaques inoculated with parental SHIV. These findings obtained in *vivo *suggest that Vpu-mediated CD4 degradation may have an important role in disease progression. However, we cannot rule-out that Vpu may have additional functions that are important for viral pathogenesis. In that regard, it is interesting to note that a recent study reported a novel activity of Vpu, that is the downregulation of CD1d at the cell surface of infected DCs and the impairment of CD1d-mediated natural killer T (NKT) cell activation [164]. CD1d is a membrane-associated protein that is expressed at the cell surface of antigen presenting cells (APCs), such as monocytes, macrophages and DCs. This protein is responsible for the presentation of exogenous pathogen-induced lipid antigens to NKT cells expressing an invariant αβT cell receptor and in doing so triggers the mutual activation of both APCs and NKT cells and the subsequent induction of cellular immune responses. Analysis of the mechanism of Vpu-mediated CD1d downregulation revealed that Vpu does not enhance constitutive endocytosis of CD1d nor induces its degradation. Rather, Vpu was found to interact with CD1d and inhibit its recycling from endosomal compartments to the plasma membrane by sequestering CD1d in an intracellular compartment. Targeting of membrane-associated surface proteins by Vpu does not appear to be limited to CD4, Tetherin and CD1d since recent evidence indicates that Vpu can also downregulate the NK-T and B cell antigen (NTB-A) co-activator at the surface of infected cells and as a result interferes with the degranulation of NK cells [165]. NTB-A is type 1 membrane-associated protein that belongs to the signaling lymphocytic activation molecules (SLAM) family of receptors that functions as a homotypic ligand-activation NK receptor pair in the induction of NK cell responses. Interestingly, NTB-A downregulation by Vpu was found to be distinct from CD4 and Tetherin downregulation since Vpu did not alter the steady-state levels of NTB-A and did not rely on the recruitment of β-TrCP to reduce cell surface NTB-A. Like with CD1d and Tetherin, Vpu did not enhance NTB-A endocytosis but rather appeared to interact with co-activator molecules through its TM domain. These findings suggest that Vpu might also downregulate NTB-A through alteration of the protein trafficking and/or recycling, which would lead to the sequestration of the molecule in an intracellular compartment. Importantly, Vpu-mediated downregulation of NTB-A was found to interfere specifically with NK cell degranulation, thus ultimately protecting infected cells from NK cell-mediated lysis.

The fact that Vpu is now reported to target, in addition to CD4, three membrane-associated cell surface proteins involved in various aspects of the innate immune response raises the possibility that Vpu may be a key factor used by HIV-1 to evade innate immunity. While it may be too soon to call Vpu a virulence factor, the recent discoveries presented in this review tend to suggest that this accessory protein provides HIV-1 with unique properties at the level of virus transmission and escape from host defenses. Further research in these areas will not only provide exciting new insight into the role of Vpu in HIV-1 pathogenesis but may also lead to new therapeutic interventions for treating HIV-1 infection.

## Competing interests

The authors declare that they have no competing interests.

## Authors' contributions

MD, MB, CP, EAC drafted sections of the text, edited each other's contributions, read and approved the final manuscript.
